# Alexithymia and emotional reactions to odors

**DOI:** 10.1038/s41598-017-14404-x

**Published:** 2017-10-26

**Authors:** Cinzia Cecchetto, Raffaella Ida Rumiati, Marilena Aiello

**Affiliations:** 1 SISSA – International School for Advanced Studies, Neuroscience Area, Via Bonomea, 265, 34136 Trieste, Italy; 2 Institute of Psychology, University of Graz, Graz, Austria; 3 BioTechMed, Graz, Austria; 4 ANVUR - Agenzia Nazionale della Valutazione del Sistema Universitario e della Ricerca, Via Ippolito Nievo, 35 – 00153 Roma, Italy

## Abstract

Alexithymia is a psychological construct characterized by deficits in processing emotional stimuli. However, little is known about the processing of odours in alexithymia, even though there is extensive proof that emotion and olfaction are closely linked. The present study is aimed at investigating how alexithymic individuals process emotions conveyed by odors. Emotional responses to unpleasant, neutral odors and clean air were collected through self-report ratings and psychophysiological measures in a sample of 62 healthy participants with high (HA), medium (MA) and low (LA) levels of alexithymia. Moreover, participants performed tests on odors identification and threshold and completed questionnaires assessing olfactory imagery and awareness. Two main results have been found: first, HA and MA groups showed altered physiological responses to odors, compared to LA, while no differences among the groups were observed in odor ratings; and second, affective and cognitive alexithymia components were differently associated with the performance on olfactory tests, skin conductance response to odors, reaction times in the rating task, and scores on olfactory questionnaires. We conclude that alexithymia is characterized by altered physiological reactions to olfactory stimuli; moreover, we stress the importance of evaluating the different alexithymia components since they affect emotional stimuli processing in different ways.

## Introduction

Alexithymia - Greek for “no words for feelings”^1^ - is a psychological construct that identifies people characterized by a marked difficulty in identifying, describing, and expressing their own emotions^2–4^. Even though alexithymia was originally described in patients with psychosomatic disorders and has been related to a broad range of psychiatric and neurological disorders, such as autism spectrum condition^5^, eating disorders^6^, multiple sclerosis^7^ and Parkinson’s disease^8^, it has been suggested that it is a relatively stable personality trait^9^ with a prevalence rate of 10% in the general healthy population^10^.

Several studies have revealed that even in healthy participants, alexithymia is characterized by deficits in the processing of emotional stimuli at the behavioral, physiological and neurobiological levels (see^11^ for a recent review). For instance, alexithymic participants, when compared to non-alexithymic participants, show a reduced affective priming with both facial expressions^12^ and emotional words^13^ (but see^14^ for a contrasting result) and they seem to be less accurate in recognizing emotional facial expressions^15^. At the psychophysiological level, several studies have reported various physiological response abnormalities. On the one hand, alexithymic individuals have been characterized as presenting less physiological reactivity: for instance, they showed smaller electrodermal responses or lower increase in heart rate when presented with emotion-provoking slides^16,17^ or negative images^18,19^. The same physiological pattern has been reported in response to stress or during stress-provoking situations^20,21^. These observations have led to the so-called *hypoarousal theory* of alexithymia. However, other studies that support the *hyperarousal theory*, have found greater electrodermal activity and higher heart rate in alexithymic compared to non-alexithymic participants in response to emotional stimuli or stress^22–24^.

At the neurobiological level, alexithymia has been associated with low reactivity to negative emotional stimuli in brain regions such as the amygdala and the insula involved in the encoding of affective stimuli^25,26^.

The investigation of the processing of emotional stimuli in alexithymic participants has involved not only the visual modality but also auditory stimuli such as prosody and music. For instance, high alexithymia scores significantly associate with worse performance on the identification of emotional prosody^27^. Moreover, participants with high alexithymia scores show significantly smaller N400 amplitudes in response to affectively incongruent music and speech targets^28^ and larger N100 amplitudes in response to emotional prosodies^27,29^.

Surprisingly, very little is known about the processing of emotions conveyed by olfactory stimuli in alexithymia, even though a strong link between emotion and olfaction has been extensively proved^30,31^. Indeed, the olfactory system is anatomically connected to the limbic system through brain regions including the amygdala, piriform cortex, insula, orbitofrontal cortex and anterior cingulate cortex^30,32,33^. This preferred link is manifested in the noteworthy ability of odors to automatically induce mood changes^31^ and to impact cognition and behavior^34–36^.

To our knowledge, olfactory perception in the alexithymia context has been investigated only in the study by Lombion, *et al*.^37^. This study included three groups of women: two groups with high and low alexithymia, as well as anorexia and depression, plus a third group of healthy controls^37^. All participants were tested for odor threshold perception and asked to rate the intensity and the hedonic valence of 13 odors. Even though the three groups did not differ in terms of odor threshold perception, they differed in self-ratings of the perceived odor intensity and valence^37^, with alexithymic patients showing higher values for intensity and pleasantness compared to controls and non-alexithymic patients. However, this study does not allow us to draw firm conclusions regarding possible deficits in emotional odor perception associated with alexithymia. First, it has been shown that both severely depressed patients^38–40^ and patients with anorexia^41^ present reduced olfactory sensitivity. Second both patient groups received medications that could affect odor perception^42^. Third, and most importantly, the delivery, selection and evaluation of odor stimuli have been partially neglected.

Therefore, in the present study we sought to investigate the association between alexithymia and emotional response evoked by odors in healthy subjects. To this end, sixty-two participants were divided into three groups in accordance with the cut-off scores proposed for the Bermond–Vorst Alexithymia Questionnaire, form B (BVAQ^43,44^): high alexithymia (HA), low alexithymia (LA) and medium level of alexithymia (MA). In contrast to the Toronto Alexithymia Scale (TAS-20), which assesses only the cognitive alexithymia dimension, the BVAQ also measures its affective dimension^43^. Recent studies^45–47^ have shown that the affective and the cognitive alexithymia dimensions may differentially affect the processing of emotions and that they are related to separate neural correlates. Importantly, it has been observed that these two components affect sympathetic responses differently: high scores in the cognitive dimension (verbalizing, identifying and analyzing) result in lower baseline response skin levels, while high scores in the affective alexithymia dimension (emotionalizing and fantasizing) have been found to be associated with higher response skin peak values in response to fear stimuli.

Odor can be independently classified according to their hedonic values (or valence^48^) and intensity (a proxy for arousal^49^). In particular, valence can be described in a continuum of pleasantness, in which negative valence represents unpleasant sensory experience and positive valence describes pleasant sensory experience^50^. Based on these mechanisms, participants were asked to perform an odor-rating task on an emotionally valenced odor, a neutral odor and clean air used as control presented through an olfactometer^51^, in which they were asked to rate the intensity, the pleasantness and the familiarity (which could be related to valence and so could be an additional variable of interest^52^) of each of the three odors, by answering the following questions: “How intense was the odor you just smelled?”, “How pleasant was the odor you just smelled?” and “How familiar was the odor you just smelled?”. Furthermore, skin conductance response (SCR) and instantaneous heart rate (IHR) were simultaneously recorded.

In addition, participants performed an odor identification test (Sniffin’ Sticks subtest^53^) and an odor threshold test. Finally, they completed the Odor Awareness Scale (OAS^54^), that assesses the awareness of odors in the environment, and the Vividness of Olfactory Imagery Questionnaire (VOIQ^55^) that estimates the olfactory mental imaging ability.

Linear Mixed-effects models (LMMs) with and without interactions between groups and odor stimuli and with only odor stimuli as factors were compared to find the best models fitting with the data. LMM were applied to odors ratings, RTs and psychophysiological measures. Based on Lombion, *et al*.^37^ and on previous evidence of atypical physiological reactivity to emotional stimuli^16–19^, differences between HA and LA were expected for odors ratings and for psychophysiological measures. Furthermore, since a recent study did not find significant differences between LA and MA in SCR recorded during classical fear conditioning (Starita, *et al*.^56^), significant differences were not expected between these two groups.

Finally, since alexithymia has been described as a personality trait that encompasses both cognitive and affective dimensions, each composed of multiple components (verbalizing, fantasizing, and identifying for the cognitive dimension; emotionalizing and analyzing for the affective component), we also explored which of these components were implicated in olfactory perception and emotional reactions to pleasant or unpleasant odors.

## Results

### Odor-rating task: behavioral responses

**Figure 1:**
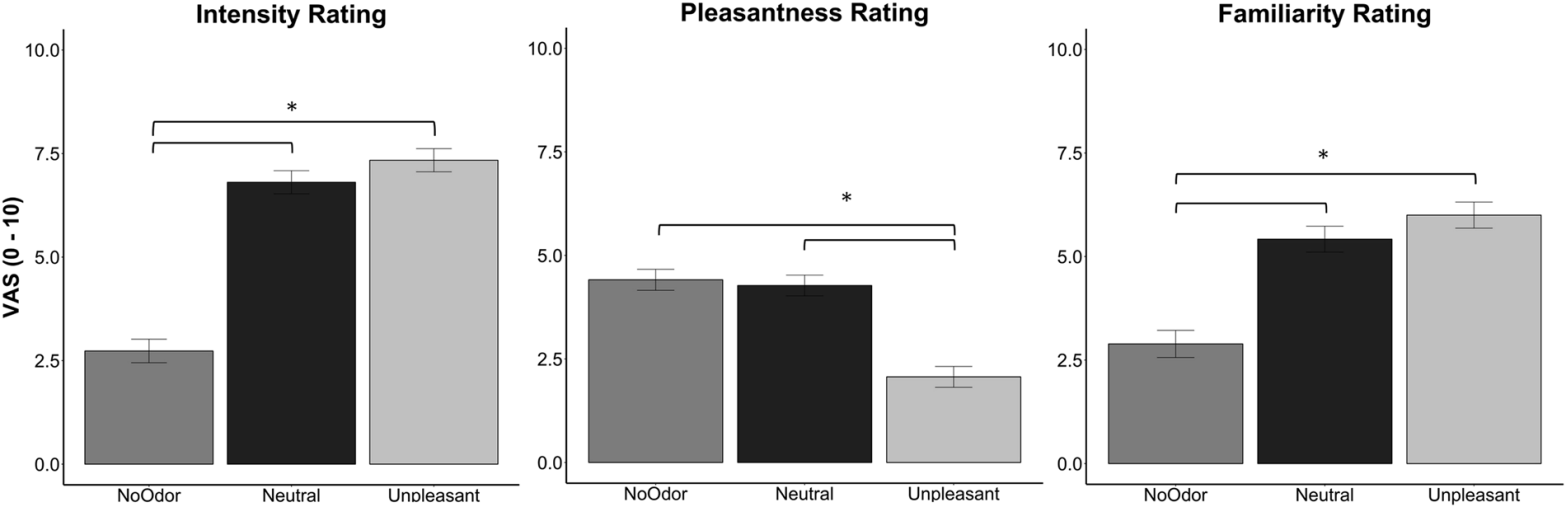
Distribution of intensity, pleasantness and familiarity ratings per odor conditions. Error bars represent the simulated 95% confidence interval of the coefficients. Significant differences are indicated.

Linear Mixed Model (LMM) with only odor factor resulted the best model (identified as the one that minimized AIC) fitting intensity, pleasantness and familiarity ratings. LMM on intensity rating (AIC = 818.74; BIC = 834.81; logLik = −404.37; R^2^ = 0.55; see Fig. 1) revealed odor as a significant factor (clean air: M = 2.73, SD = 2.00; neutral odor: M = 6.81, SD = 2.12; unpleasant odor: M = 7.34, SD = 2.51): clean air was perceived as less intense than the other odors (all ß > 2.74, all SE < 0.38, all *t* > 9.53, all *p* < 0.001) but no significant difference was found between neutral and unpleasant odors (ß = 0.53, SE = 0.37, *t* = 1.45, *p* = 0.15). The LMM model including the interaction group*odor factors showed no significant differences between groups and no significant interactions.

LMM on pleasantness rating (AIC = 776.39; BIC = 792.44; logLik = −383.19; R^2^ = 0.28) revealed odor as a significant factor (clean air: M = 4.42, SD = 2.06; neutral odor: M = 4.27, SD = 2.17; unpleasant odor: M = 2.07, SD = 1.69; see Fig. 1): unpleasant odor was perceived as less pleasant than clean air (ß = 2.34, SE = 0.34, *t* = 6.71, *p* < 0.001) and neutral odor (ß = 2.21, SE = 0.35, *t* = 6.37, *p* < 0.001) but no significant differences were found between clean air and neutral odor (ß = 0.14, SE = 0.34, *t* = 0.39, *p* = 0.69). The LMM model including the interaction group*odor factors showed no significant differences between groups and no significant interactions.

LMM on familiarity rating (AIC = 839.42; BIC = 855.36; logLik = −414.71; R^2^ = 0.28) revealed odor as a significant factor (clean air: M = 2.88, SD = 1.90; neutral odor: M = 5.42, SD = 2.24; unpleasant odor: M = 6.00, SD = 3.11; see Fig. 1): clean air was perceived as less familiar than the other odors (all ß > 2.52, all SE < 0.44, all *t* > 5.72, all *p* < 0.001) but no significant differences were found between neutral and unpleasant odors (ß = 0.58, SE = 0.43, *t* = 1.43, *p* = 0.18). The LMM model including the interaction group*odor factors showed no significant differences between groups and no significant interactions. See Table 1S in Supplemental Information for means and standard deviations of odor-ratings for groups and odors. See Supplemental Information for results of reaction times analysis.

### Odor-rating task: SCR

**Figure 2:**
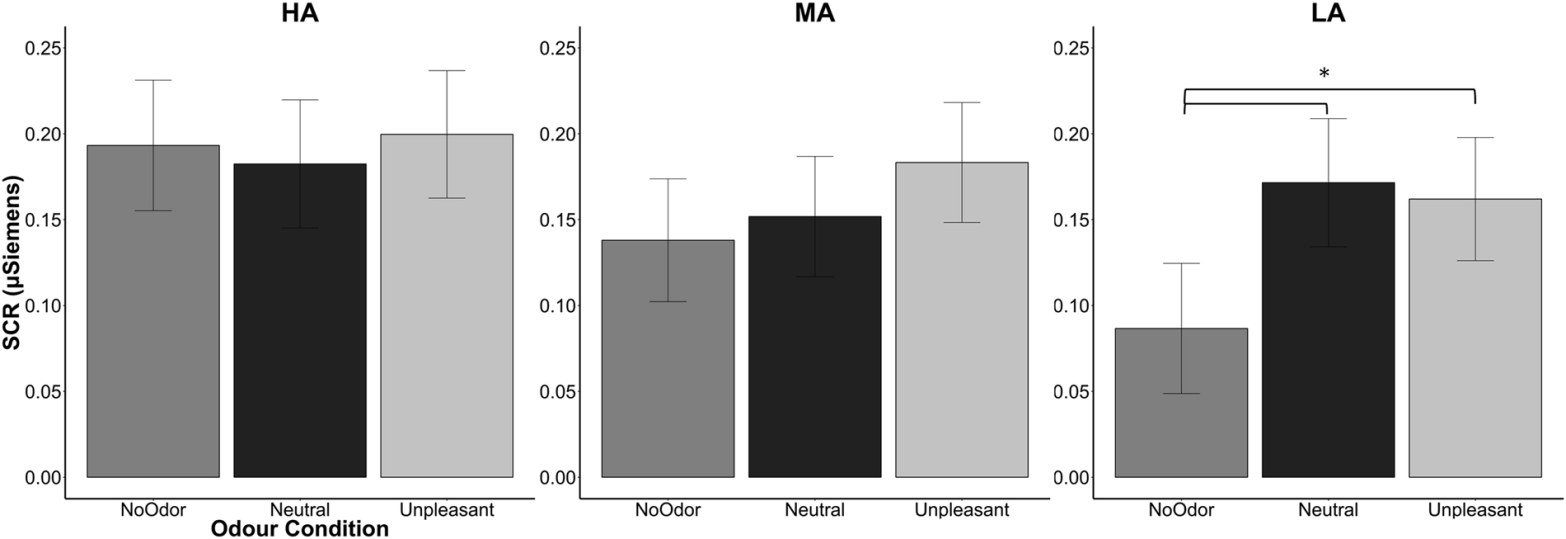
SCR per groups and odor conditions. Error bars represent the simulated 95% confidence interval of the coefficients. Significant differences are indicated. SCR = skin conductance responses; HA = high level of alexithymia; MA = medium alexithymia; LA = low alexithymia.

LMM with interaction between odor and group resulted the best model fitting SCR (AIC = −251.85; BIC = −207.28; logLik = 136.93; R^2^ = 0.38; see Table 1 for *β*, *t* and *p* values). The analysis revealed that HA group showed greater SCR than LA group (*p* = 0.04). Moreover, a significant interaction between LA group and neutral odor was found (*p* = 0.047), however the post-hoc analysis (*lsmeans* function) showed not significant results. To better analyze the significant result of group factor (see Fig. 2), LMM with odor as only factor was applied separately for each group. LA group showed greater SCR for neutral (ß = 0.08, SE = 0.04, *t* = 2.29, *p* = 0.002) and unpleasant odors (ß = 0.07, SE = 0.03, *t* = 2.12, *p* = 0.04) compared to clean air. No significant differences were found between neutral and unpleasant odors (ß = 0.009, SE = 0.03, *t* = 0.28, *p* = 0.78). No significant differences were found between odors for HA (all ß < 0.006, all SE > 0.03, all *t* < 0.19, all *p* > 0.75) and for MA (all ß < 0.04, all SE > 0.02, all *t* < 1.51, all *p* > 0.13). Similar results were also found after controlling for depression scores (Beck Depression Inventory, BDI^57^; see results in the Supplemental Information). However, when we run the analysis separately for each group, we found that BDI had an opposite effects in the LA and HA groups: in the LA group the more participants were depressed the greater the SCR, for in the HA group the more participants were depressed the weaker the SCR (see Supplementary Information for more details).

**Table 1:** Summary of the best fitting LMM for SCR.

SCR	*β*	*SE*	*t value*	*p value*
*Fixed effects*				
Group *(MA)*	−0.05	0.05	−1.03	0.30
Group *(LA)*	−0.11	0.05	−2.06	0.04
Odor *(Neutral)*	−0.011	0.03	−0.34	0.73
Odor *(Unpleasant)*	0.006	0.03	0.20	0.84
Group*Odor (MA*Neutral)	0.02	0.04	0.52	0.60
Group*Odor (LA*Neutral)	0.09	0.04	1.98	0.05
Group*Odor (MA*Unpleasant)	0.04	0.05	0.84	0.40
Group*Odor (LA*Unpleasant)	0.07	0.05	1.48	0.14

*Note: β* = estimate; SE = standard error; 95%; MA = medium alexithymia; LA = Low alexithymia. Significant p values are in bold. Reference condition for categorical factors is reported in italic inside bracket.

### Odor-rating task: IHR

LMM with interaction between time windows and group resulted the best model fitting IHR (AIC = 10818.42; BIC = 10877.84; logLik = −5398.212; R^2^ = 0.18; see Table 2 for *β*, *t* and *p* values). The analysis showed a significant effect of time window: IHR significant decelerated during the second (*p* < 0.001) and third (*p* < 0.001) time windows compared to the first time window which corresponds to odor presentation, and it presented a significant acceleration during the third time window compared to the second one (*p* = 0.02). Furthermore, the LA group presented a greater IHR deceleration than MA group (*p* = 0.02). Finally, the interaction time window* group resulted significant but the post-hoc analysis showed no meaningful significant results.

**Table 2:** Summary of the best fitting LMM for IHR.

IHR	*β*	*SE*	*t value*	*p value*
*Fixed effects*				
Time window *(2)*	−3.58	0.66	−5.41	<0.001
Time window *(3)*	−2.08	0.66	−3.15	<0.001
Group *(LA)*	−1.57	0.97	−1.61	0.112
Group *(MA)*	0.73	0.99	0.74	0.459
Group* Time window (LA*2)	0.82	0.93	0.87	0.013
Group* Time window (LA*3)	1.55	0.93	1.66	0.018
Group* Time window (MA*2)	−2.36	0.95	−2.49	0.012
Group* Time window (MA*3)	−2.24	0.95	−2.36	0.018

*Note: β* = estimate; SE = standard error; 95%; BDI = Beck Depression Inventory; MA = medium alexithymia; LA = Low alexithymia. Significant p values are in bold. Reference condition for categorical factors is reported in italic inside bracket.

**Figure 3:**
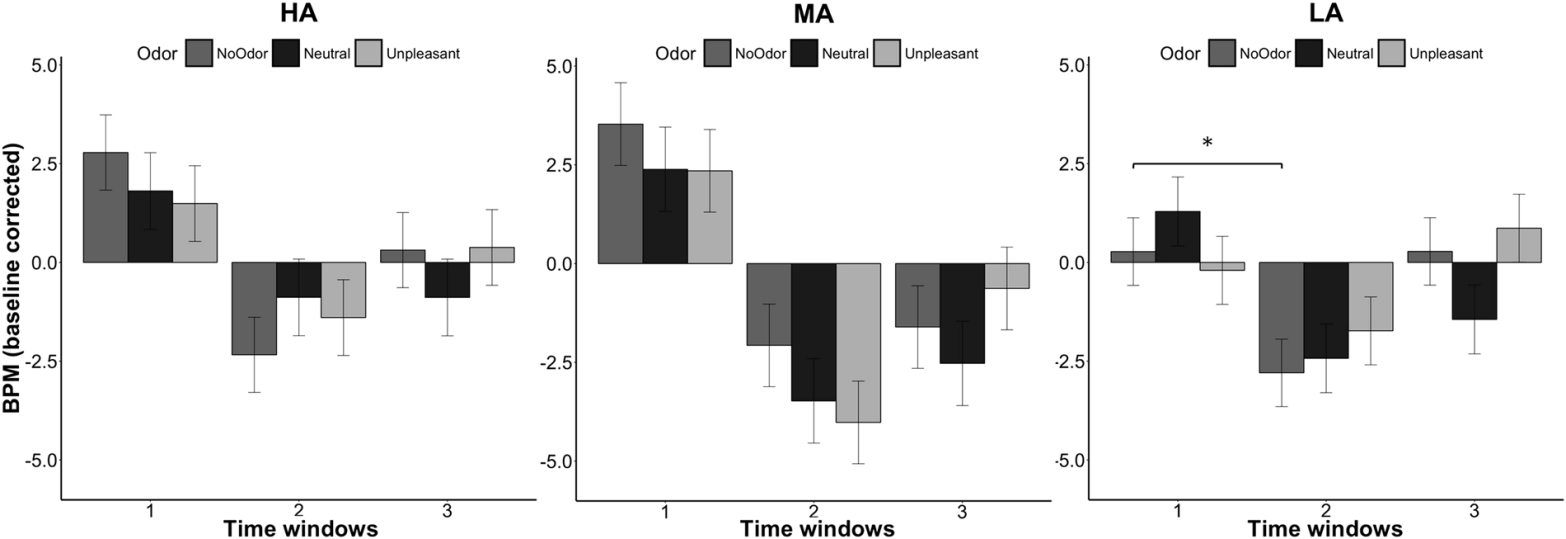
IHR per groups, time windows and odor conditions. Error bars represent the simulated 95% confidence interval of the coefficients. Significant differences are indicated. IHR = instantaneous heart rate; HA = high level of alexithymia; MA = medium alexithymia; LA = low alexithymia.

As for the SCR analysis, LMM with interaction between time windows and odor was applied separately on each group to better investigate differences in odor conditions (see Fig. 3). HA group showed greater IHR acceleration during time window 1 compared to time windows 2 (ß = −2.69, SE = 1.24, *t* = −2.16, *p* = 0.03) and 3 (ß = −2.69, SE = 1.24, *t* = −2.16, *p* = 0.03) but no effects of odors or significant interactions were found. Even MA group showed a significant time window effect, greater IHR acceleration during time window 1 compared to time windows 2 (ß = −5.86, SE = 1.17, *t* = −5.00, *p* < 0.0001) and 3 (ß = −4.91, SE = 1.17, *t* = −4.19, *p* = 0.0001), but no significant odors effect or significant interactions were found. LA group revealed the same significant effect of time window: greater IHR acceleration during time window 1 compared to time windows 2 (ß = −3.71, SE = 1.10, *t* = −3.36, *p* < 0.001) and 3 (ß = −2.73, SE = 1.10, *t* = −2.47, *p* = 0.014). Moreover, significant interaction was found between unpleasant odor and time window 3 (ß = 3.79, SE = 1.54, *t* = 2.45, *p* = 0.014) while post-hoc analysis showed a significantly greater acceleration between clean air condition in time window 1 compared to clean air condition in time window 2 (*p* = 0.023). Similar results were also found after controlling for depression scores (see results in the Supplemental Information).

### Olfactory screening: Identification and threshold tests

The LMM with group as a factor on the identification test showed that group is a significant factor, indeed HA presented significantly lower accuracy compared to MA group (ß = 0.74, SE = 0.37, *t* = 2.00, *p* = 0.049) but not compared to LA group (ß = 0.57, SE = 0.37, *t* = 1.55, *p* = 0.12). However, when the BDI score is included, the LMM showed no more significant effects of group (all ß < 0.70, all SE > 0.37, all *t* < 1.82, all *p* > 0.07) or BDI (ß = −0.01, SE = 0.02, *t* = −0.78, *p* = 0.43). For the threshold test, similar non-significant results were obtained for LMM only with group and with BDI (all ß < 0.18, all SE > 0.02, all *t* < 0.86 all *p* > 0.39). There was no main effect for the BDI factor.

### Subjective reports of odor awareness and olfactory imagery

LMMs with the group factor alone or with group and BDI as factors showed no significant results when applied on both OAS data (all ß < 7.21, all SE > 5.43, all *t* < 1.33, all *p* > 0.19) and VOIQ data (all ß < 7.61, all SE > 5.85, all *t* < 1.30, all *p* > 0.19).

### Analysis of BVAQ subcomponents

To explore the possible role of the five components of alexithymia on emotional response evoked by odors and on olfactory sensitivity LMMs with the five subcomponents of BVAQ were conducted on intensity, pleasantness, familiarity ratings, reaction times, SCR and IHR of odor-rating task, on olfactory screening tests (identification and threshold) and personality differences (OAS and VOIQ). B1 (*verbalizing*) resulted as a significant predictor for RT of pleasantness rating (ß = −65.46, SE = 24.52, *t* = −2.69, *p* = 0.009) and familiarity rating (ß = −66.06, SE = 26.85, *t* = −2.462, *p* = 0.017): the higher the score on the verbalizing scale the shorter the RTs.

**Figure 4:**
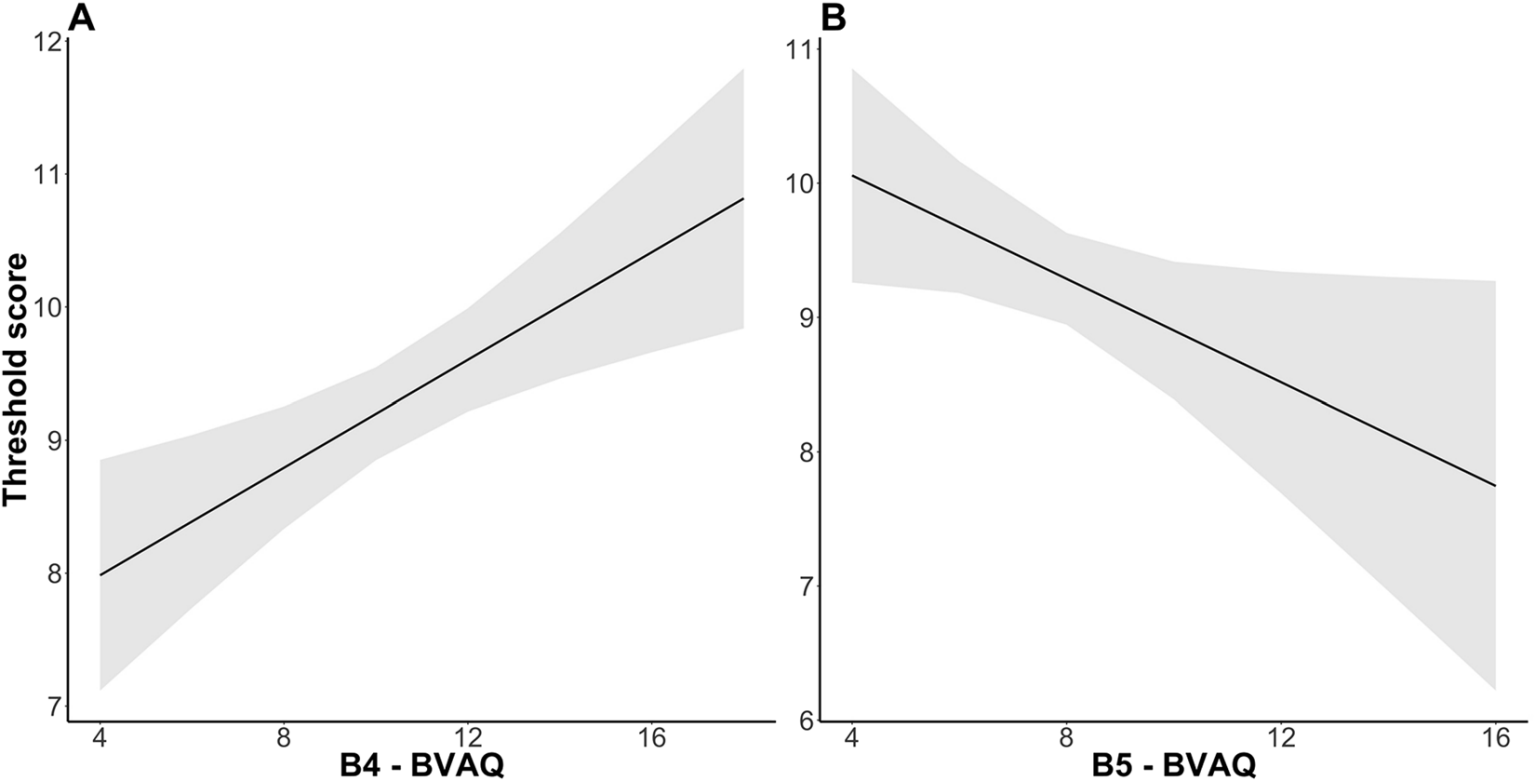
(**A**) Effect of B4 and (**B**) of B5 components on Threshold test score. Grey areas represent the simulated 95% confidence interval of the coefficients. B4 = emotionalising component of Bermond–Vorst Alexithymia Questionnaire; B5 = analysing of Bermond–Vorst Alexithymia Questionnaire.

**Figure 5:**
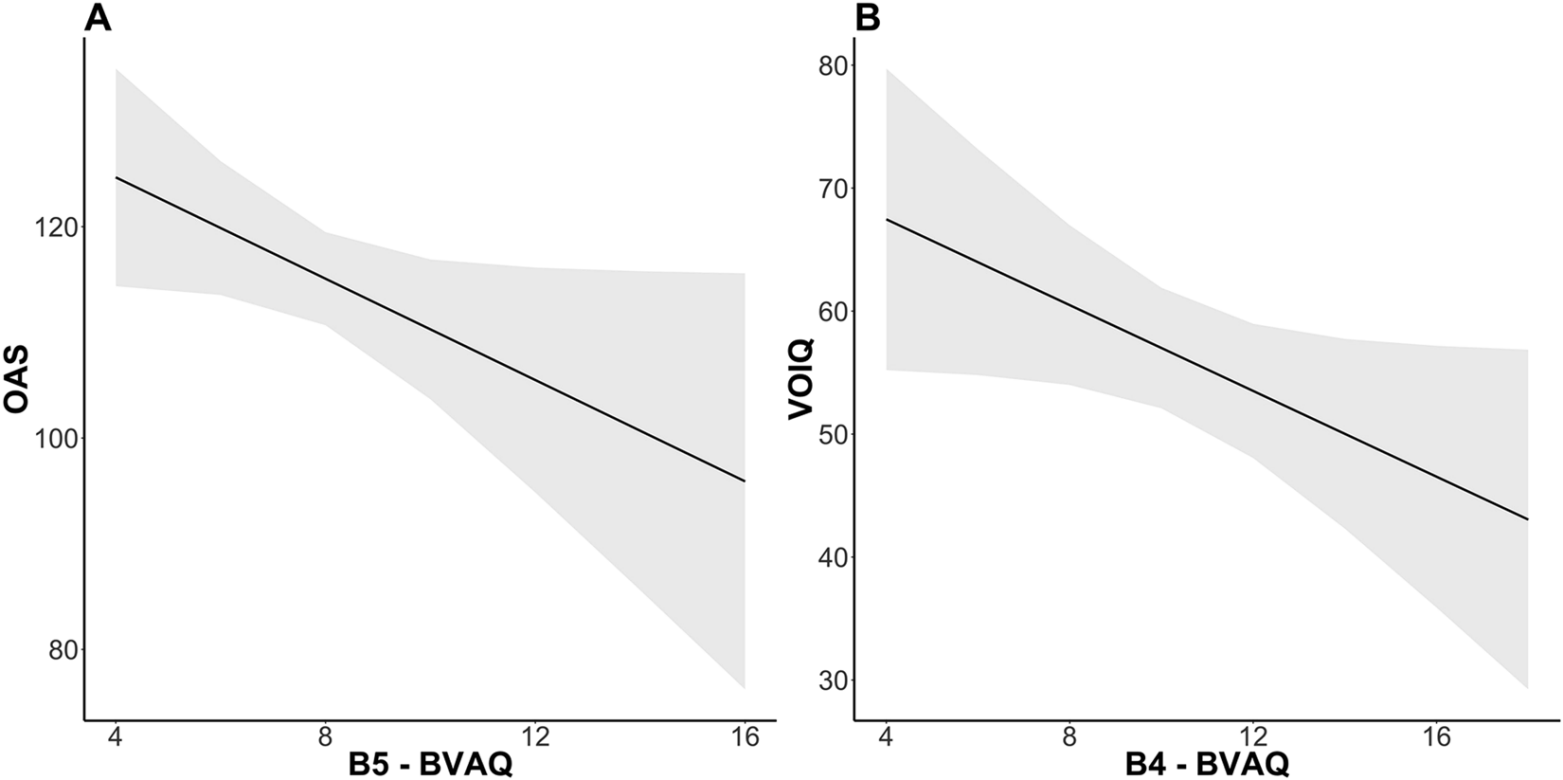
(**A**) Effect of B5 component on OAS questionnaire; (**B**) Effect of B4 component on VOIQ questionnaire. Grey areas represent the simulated 95% confidence interval of the coefficients. OAS = Odor Awareness Scale; VOIQ = Vividness of Olfactory Imagery Questionnaire; B4 = emotionalising component of Bermond–Vorst Alexithymia Questionnaire; B5 = analysing of Bermond–Vorst Alexithymia Questionnaire.

B3 (*identifying*) resulted as a significant predictor for SCR (ß = 0.018, SE = 0.007, *t* = 2.63, *p* = 0.001), the higher the score on this scale the greater SCR. For what concerns IHR, no significant effects were found for BVAQ components alone or in interactions with the odor conditions. Furthermore, B4 (ß = 0.20, SE = 0.06, *t* = 3.31, *p* = 0.002) and B5 (*analyzing;* ß = −0.19, SE = 0.09, *t* = −2.09, *p* = 0.004) were significant factors for threshold test: the higher the score in analyzing scale the higher the odor threshold and the higher the score on the emotionalizing scale the lower the odor threshold (so the better the odor sensitivity; see Fig. 4). Moreover, B5 resulted as a significant predictor for OAS (ß = −2.39, SE = 1.18, *t* = −2.02, *p* = 0.048): the higher the score on the emotionalizing scale the worse the odor awareness; and B4 resulted a significant predictor for VOIQ (ß = −1.74, SE = 0.86, *t* = −2.01, *p* = 0.048): the higher the score on the analyzing scale the worse the vividness of olfactory imagery (see Fig. 5). No significant results were found for the B2 component (*fantasizing*).

## Discussion

In the present study we examined the differences between HA, MA and LA individuals in processing emotional olfactory stimuli. To this end, emotional responses to unpleasant odor, neutral odor and clean air as control were collected through self-report ratings and psychophysiological measures. Moreover, odor identification and threshold tests were administered and several questionnaires were completed as measure of olfactory sensibility and odor awareness, respectively. Two main results have been found. First, the presence of alexithymia resulted associated with altered physiological responses to odors, despite no differences compared to non-alexithymics were observed in general odor ratings. Second, different components of alexithymia resulted associated with the performance in olfactory tests, psychophysiological responses to odors, RT in the rating task and scores at VOIQ and OAS.

Previous literature has suggested that alexithymic participants present a decoupling between subjective experience and psychological responses^19,23,56^, which may explain why many studies^19,23,56,58–60^ failed to find behavioral differences between high and low alexithymics when compared in cognitive tasks or explicit emotional conditions. Consistently with this idea, Starita, *et al*.^56^ found comparable subjective reports of fear and anxiety between HA, LA and MA during the expectation and the presentation of a fear conditioning event, despite HA participants presented lower physiological response compared to LA and MA. Moreover, Eastabrook, *et al*.^23^ showed that although alexithymic males exhibit hyper-arousal during a spontaneous speech test, they express great self-consciousness.

Our results are in line with this evidence: participants with HA behaved as participants with LA when they had to subjectively report the intensity, pleasantness and familiarity of odors. However, differently from LA individuals who presented significant greater SCR for neutral and unpleasant odors compared to no odor condition (which corresponds to the orientation towards newly introduced stimuli^61^), HA individuals presented greater SCR for all three odor conditions independently of the presence/absence of an odor. A similar pattern of results was found also in IHR analysis: compared to the LA group, the HA present a greater acceleration in time window 1 (during odor presentation) but they did not show any odor effect on time window 3, which instead is slightly present in the LA group. These results are not consistent with the hypothesis of an attenuated affective reactivity or a condition of hypoarousal^47,56,62^ and are more in line with the hyperarousal theory of alexithymia, which advocates that alexithymics display exaggerated physiological responses to emotional stimuli^23,24,63^. Indeed several studies have pointed out conflicting results when physiological measures have been considered in alexithymics. These conflicting results can be explained by the different experimental tasks or stimuli being used. In this regard, we should also mention that, to our knowledge, this is the first study in which psychophysiological responses to odor stimuli have been investigated in alexithymic participants.

Contrary to our expectations and unlike Starita, *et al*.^56^, we found that MA behaves as HA in psychophysiological measures. This result suggests that individuals with medium levels of alexithymia may show affective abnormalities and that this group should be enrolled in studies on alexithymia.

As in the study proposed by Lombion, *et al*.^37^, the three groups in this study did not present significant differences in odor perception as measured by standardized olfactory tests. However, in contrast with our data, the previous study^37^ showed that alexithymic individuals exhibited higher intensity and hedonic ratings for odors compared to the other two groups. Different aspects could explain this contrasting result. Unlike our study, in Lombion, *et al*.^37^ participant sample included patients with eating disorders and under medication. Most importantly, these authors did not control for BDI scores in their participants. Finally, the three groups had significant differences in anhedonia, which may affect olfactory hedonics^64^.

In line with previous studies^38–40^, our data indicated that depression scores could affect results related to olfactory processing: indeed, differences in the identification task between HA and MA groups resulted no more significant when depression scores were controlled. This is an important confirmation of the importance of controlling for depression in studies that investigate emotional processing in alexithymia. Moreover, our results also highlighted that depression may exert a different effect on psychophysiological measures according to the presence or absence of alexithymia, an aspect that deserves more investigation.

As suggested in a recent review by Donges and Suslow^11^, it is important to consider the different components of alexithymia while investigating how emotional stimuli are processed. Our results support previous evidence showing that different components of alexithymia (emotionalizing, verbalizing, identifying and analyzing) seem to be associated with different aspects of olfactory processing. The emotionalizing component (B4) turned out to be a significant predictor of the threshold test and the VOIQ questionnaire, which assesses the individual differences in odor imagery ability^55^. This component has been described as “the degree to which someone is emotionally aroused by emotion inducing events”^65^. Participants who are emotionally less aroused by emotional events also show weaker olfactory sensibility and olfactory imagery. This evidence might support the hypothesis that the impaired automatic emotional processing of alexithymic participants could affect their olfactory processing^66^. For example, previous studies have shown that the presentation of unpleasant pictures significantly decreases olfactory sensitivity^66^.

The verbalizing component (“the degree of the ability or the disposition to describe or communicate about her emotional reactions”^65^) significantly predicted reaction times: the more participants presented difficulties in communicating their emotions, the faster they performed on odor-rating task. Moreover, the higher the score in the identifying component (“the degree to which one is able to define one’s arousal states”^65^), the greater the SCR. Verbalizing and identifying are both included in the cognitive alexithymia dimension. Bermond, *et al*.^47^ suggested that individuals with reduced emotional cognitive capacities ruminate less than those with high emotional cognitive capacities, and they present reduced physiological activations^67^. Our results only partially support this hypothesis; indeed, participants who scored higher in components of cognitive alexithymia seem to think less about their answers even though they have a stronger SCR response.

Finally, we found that the analyzing component, which was described as “the degree to which one seeks out explanations of one’s own emotional reactions”^65^, significantly affected threshold and OAS scores. This result is quite unexpected; at the same time, participants with higher difficulties in explaining their emotions seem to present a lower odor threshold, and therefore higher odor sensibility, but they seem to be less aware of the odor presented in the environment. This contradictory result could be related to the different nature of the two tests (one is an implicit odor threshold test, the other a self-report questionnaire) and so participants could have used different strategies to complete the tests. Future studies should clarify this aspect.

It is interesting to notice that our participants overall rated clean air as significantly less intense and less familiar than the other odors but with the same level of pleasantness than the neutral odor. Moreover, unpleasant odor was perceived as significantly more unpleasant than the other two odor conditions and participants were significantly faster when they had to decide pleasantness of the unpleasant odor compared to the other odors, as expected in the rating of an unpleasant stimulus^68^.

Finally, we are aware that our research may have some limitations. First, the number of trials is quite small. This experimental paradigm was design to avoid repetition of the odor conditions and the related adaptation effect in psychophysiological responses and the distortion of familiarity responses. However, future studies could use same odors but disparate odor concentrations. Second, in the present experiment we used a self-report questionnaire to measure alexithymia. Several criticisms have been raised regarding the use of these measures to assess alexithymia since alexithymic individuals are not aware of their emotional impairments. Future studies should try to insert in their experimental design the structured interview for the Diagnostic Criteria for Psychosomatic Research to confirm the presence or absence of alexithymia^69^. Finally, in accordance with participants’ ratings, we used gardenia as a neutral odor, despite it is often used as a pleasant odor in the literature (for instance see^70^). Future studies should evaluate olfactory processing in alexithymia using neutral, pleasant and unpleasant olfactory stimuli.

In conclusion, the present study points out that in healthy subjects alexithymia is associated with altered autonomic emotional reactions to olfactory stimuli. These results seem to suggest that the difficulties described in alexithymic individuals in processing visual and auditory emotional stimuli are extended also to olfactory emotional stimuli. Moreover, since olfactory loss has been associated with a reduction of quality of life^71,72^ and depression^38,39^, further investigations are needed to explore possible relations between olfactory abnormalities and the strong relation between alexithymia and depression^73,74^. Finally, the analysis of alexithymia components revealed a distinct pattern of olfactory abnormalities related to each single component. Further clarification and characterizations of these olfactory aspects could help to examine the mechanism behind alexithymia and to guide the realization of future assessments.

## Materials and Methods

### Participants

An initial sample of 502 people, mainly students of the University of Trieste, was contacted through social network and word of mouth. From this sample, 62 participants were selected. The exclusion criteria were: history of neurological or psychiatric disorders, being a smoker, using psychopharmacological substances, having experienced head trauma leading to unconsciousness, score less than 10 at the Sniffing Sticks Identification test^53^. Sample size and data collection stopping rule were based on sample and effect size reported in literature on emotion processing in alexithymia^11,18^.

Participants were divided into three groups based on the BVAQ proposed cut-off scores^44,46^: LA (BVAQ score <43); MA (43 ≤BVAQ ≤52) and HA (BVAQ >52). The three groups did not differ for age [*F* (2, 55) = 0.86, *p* = 0.32], number of females [*X* ^2^(2) = 4.15, *p* = 0.12] and the BDI^57^ scores [*F* (2, 55) = 1.46, *p* = 0.24). Please, refer to Table 3 for details on the participants’ characteristics. The SISSA Ethics Committee approved the study, which is in accordance with the Declaration of Helsinki and an informed written consent was obtained from each participant.

**Table 3:** Summary Table of Demographic Characteristics and Questionnaires of groups.

	HA	MA	LA
	Mean (SD)	Mean (SD)	Mean (SD)
Sample	21	20	21
F:M	17:4	12:8	18:3
Age	22.94 (4.21)	23.74 (2.66)	24.8 (2.63)
BDI	12.26 (6.82)	9.37 (7.45)	8.75 (6.09)
BVAQ	57.52 (6.54)	48.21 (2.66)	37.15 (4.88)
B1	13.90 (2.74)	12.30 (3.21)	8.28 (2.53)
B2	11.14 (2.61)	8.80 (2.55)	7.62 (2.78)
B3	10.95 (2.76)	8.35 (2.39)	7.09 (1.94)
B4	12.33 (3.35)	10.75 (2.79)	8.48 (1.83)
B5	10.19 (2.33)	7.85 (1.42)	5.71 (1.58)
TAS-20	51.95 (12.16)	43.15 (13.59)	34.76 (6.95)
Identification test	12.90 (1.26)	13.65 (1.49)	13.47 (0.68)
Threshold test	9.22 (1.77)	9.41 (1.16)	9.27 (1.27)
OAS	115.51 (13.55)	111.45 (16.82)	118.67 (20.95)
VOIQ	51.04 (12.20)	58.8 (13.07)	58.67 (27.41)

*Note*: HA = high alexithymia; MA = medium alexithymia; LA = Low alexithymia; SD = standard deviation; M = male; F = female; BDI = Beck Depression Inventory; BVAQ = Bermond–Vorst Alexithymia Questionnaire; B1 = verbalizing component of Bermond–Vorst Alexithymia Questionnaire; B2 = fantasizing component of Bermond–Vorst Alexithymia Questionnaire; B3 = identifying component of Bermond–Vorst Alexithymia Questionnaire; B4 = emotionalizing component of Bermond–Vorst Alexithymia Questionnaire; B5 = analyzing of Bermond–Vorst Alexithymia Questionnaire; TAS-20 = OAS = Odor Awareness Scale; VOIQ = Vividness of Olfactory Imagery Questionnaire.

### Self-report Questionnaires

During the screening procedure participants were asked to complete the BVAQ^43^ designed to measure the five dimensions of alexithymia: emotionalizing, fantasizing, identifying, analyzing and verbalizing^65^.

During the experimental session participants completed the TAS-20 (Toronto Alexithymia Scale 20 items^75^), to increase the reliability of the BVAQ screening, in addition to OAS^54^ and VOIQ^55^. See Supplemental Information for description of self-report questionnaires.

### Olfactory screening

Odor identification ability was determined with the Identification subtest of Sniffin’ Sticks battery^53^. Participants were asked to identify 16 odorants from a list of four choices. Odorants were presented for about 3 s and participants could smell each odor as often as needed. The final score was the sum of the identified odors^53^.

Detection threshold was determined using a two-alternative forced-choice ascending staircase paradigm with seven reversals and no feedback^36^. Each trial included one target (a bottle with the diluted odor) and one control stimulus (a bottle with the diluent only). The odor was presented in ascending concentrations until the participant discerned correctly the odor in two successive trials, which triggered a reversal. Mineral oil (Sigma-Aldrich, Italy) was used as the diluting agent to create all the concentrations of the odor threshold test. The dilution series was prepared starting from 12.5% volume to volume (v/v) in liquid phase of cedarwood oil. From there, the odor was diluted in 16 consecutive dilution steps using a 0.5 volume dilution series (end concentration 0.00038%). Two series of the same odor were prepared and used to allow the odor to saturate the headspace between potential repetitions of the same dilution step. Each dilution step and the matched diluent only were delivered using amber 2-oz glass bottles, all visually identical and containing 10 mL of liquid each. Detection threshold was defined as the geometric mean of the last four reversals.

### Odor-rating task

The odor-rating task consisted of the presentation of three odor stimuli: unpleasant odor (butyric acid, 0.001% v/v in propylene glycol; Sigma-Aldrich, Italy), neutral odor (gardenia essential oil, 0.03% v/v in propylene glycol; Aroma-Zone, France) and clean air, meaning the absence of an odor, reproduced by an empty cleaned jar, used as control. For the odor selection procedure see Supplemental Information.

The odor-rating task was composed of 3 blocks (one block per odor condition), each block included 3 trials. Each trial began with a white screen for 4 s followed by a black and green crosses displayed respectively for 6 s (jittered) and 0.5 s. Subsequently, the odor was presented for 4 s, followed by the question screen. Ratings were collected on a 10-cm computerized VAS, ranging from “not at all” to “very much.” Participants were instructed to answer even if they did not perceive any odor within a 6-s time window/rating. The order of blocks and trials were randomized across participants, with a 1 min break between blocks.

Odors were presented birhinally using a computer-automated olfactometer to deliver odors in a temporally precise manner^51^. A low birhinal flow rate of 3.0 L/m (a total of 1.5 L/m per nostril) was used to prevent irritation of the nasal mucosa over time^51^. Following each odor presentation, while the question slide was presented clean air was introduced to minimize odor residuals^76^.

### Procedure

Upon arrival, each participant was seated in a quiet room and asked to complete the self-report questionnaires, the odor identification test^53^ and the odor detection threshold test. Afterwards, participants were asked to sit in front of a computer screen with electrodes attached for HR and SCR recording. Following a 10-min adaptation period, psychophysiological measures were recorded during a 1-minute baseline and throughout the odor-rating task.

### Psychophysiological Data Acquisition and Analysis

SCR and HR were recorded during the olfactory rating task with a *PROCOMP infiniti system* (Thought Technology, Montreal, Canada). After a 10-minute adaptation period, and before starting the task, one minute of baseline was recorded.

SCR was measured according to guidelines^77,78^, using two 8 mm Ag/AgCl electrodes, attached to the medial phalanx surfaces of the index and ring finger of the left hand. Conductive gel was used to reduce impedance. The electrode pair was excited with a constant voltage of 0.5 V and conductance was recorded using a DC amplifier with a low-pass filter set at 64 Hz. A photoplethysmographic probe (3.2 cm/1.8 cm, photodetector LED type), placed on the middle finger of the non-dominant hand was used to assess HR at a sample rate of 2048 Hz. SCR and HR data were analyzed with Matlab using in-house scripts partially using the EEGLAB toolbox (http://sccn.ucsd.edu/eeglab/60).

SCR data were filtered with a 10 Hz low-pass filter and epoched over the 12 seconds after odor presentation. The two seconds before the scenario-screen presentation served as baseline. The *peak amplitude* was analyzed: it is defined as the difference in μSiemens between the mean value during baseline and the peak after stimulus onset. Trials with peak amplitudes below 0.01 μSiemens were excluded from the SC analysis and peak amplitudes were log-transformed to improve interpretability.

HR data were filtered with a 1 Hz high-pass filter and resampled to 256 Hz. Beat detection was performed automatically, verified visually, and corrected, if necessary. Frequency was computed as beats per minute (bpm). The 12 seconds from the odor presentation were divided in 3 time windows of 4 seconds. Interbeat intervals were computed, transformed to HR values and averaged for each 4-second window. Each time window was then corrected by subtracting the 2 seconds before scenario presentation to obtain the instantaneous heart rate (IHR^79^).

### Statistical Analysis

Analyzes were performed with LMM with intercepts for participants as *random effect* to account for the high variability across individuals. This analysis reduces Type I errors and makes possible the generalization of findings to other samples of participants^80^. LMMs were fitted and analyzed using R (version 2.10.1; http://www.r-project.org/) and in particular using *lme* function from the *nlme* package (https://cran.r-project.org/web/packages/nlme/nlme.pdf).

For the analysis of scores on olfactory screening tests (identification and threshold) and personality differences (OAS and VOIQ) LMMs were built with only groups as fixed effect factor. For the odor-rating task, we compared different LMMs for each dependent variable (intensity, pleasantness, familiarity ratings, reaction times, SCR and IHR), with and without interactions between groups and odors and with only odors as factor, to find the best models to fit with the data (as tested by the Akaike Information Criterion (AIC) test resulting from the use of the generic *anova* function). For post-hoc comparisons of significant interactions the *lsmeans* package was used. As a measure of goodness of fit of the chosen LMMs, we also report conditional

R2, which describes the proportion of variance explained by both the fixed and random factors^81^. Even though the three groups did not show significant differences in BDI scores, BDI was included together with Group factor in LMMs to control for the slightly differences on depression levels between groups.

To investigate the role played by the five alexithymia components in the processing of olfactory stimuli, LMM with participants as random effect and the five BVAQ subscales as fixed effects was applied to each dependent variable. Outliers with respect of reaction times were removed according to the outlier-labelling rule^82^. From a starting number of 744 trials, 15 trials were removed because of no response (N = 15/744, 2%) while 5 trials were removed because of long choice reaction times (>5.6 s; N = 5/729, 0.68%). Final behavioral and IHR analyzes were run on 724 trials distributed over conditions. For the SCR analyzes, data from four subjects were removed because non-responders. Of the remaining 696 trials, 134 were removed due to SCR peak amplitude lower than 0.01 μSiemens (N = 139/696, 19.9%), mostly due to habituation issues^78^. Final analyzes were run on 557 trials distributed over conditions. Pearson’ s correlations were performed between BVAQ and TAS-20 to investigate the relationship between alexithymia questionnaires and its subcomponents: see Supplemental Information.

### Data Availability

The datasets generated during and/or analyzed during the current study are available in the Supplemental Information.

## Electronic supplementary material

Supplementary Information

Supplementary Information

Supplementary Information
