# Supplementary material for: Alexithymia and emotional reactions to odors
Source: Sci Rep. 2017 Oct 26;7:14097. doi: 10.1038/s41598-017-14404-x (PMC5658372; doi:10.1038/s41598-017-14404-x)
Supplement: Supplementary file 1 — Supplementary Information [file 41598_2017_14404_MOESM1_ESM.pdf]

Alexithymia and emotional reactions to odors

Cinzia Cecchetto[1, 2, 3*], Raffaella Ida Rumiati[1, 4], Marilena Aiello[1]

[1] SISSA – International School for Advanced Studies, Neuroscience Area, Via Bonomea, 265, 34100 Trieste, Italy
[2] Institute of Psychology, University of Graz, Graz, Austria
[3] BioTechMed, Graz, Austria
[4]ANVUR - Agenzia Nazionale della Valutazione del sistema Universitario e della Ricerca, Via Ippolito Nievo, 35 - 00153 Roma, Italy

Supplemental material

Self-report Questionnaires

BVAQ. The BVAQ consists of 20 items rated on a five-point scale with total scores ranging from 20 to 100; participants with a score above 53 are considered alexithymic. The BVAQ subscales produce two dimensions: the affective dimension comprises the subscales emotionalizing and fantasizing, while the cognitive dimension comprises the subscales verbalizing, identifying and analyzing[1].

TAS-20. The TAS-20 consists of 20 items rated on a five-point scale with total scores ranging 20 to 100. It includes three subscales: Difficulty in Identifying Feelings (F1), Difficulty in Communicating Feelings (F2) and Externally Oriented Thinking (F3). The international cut-off values are the following: 20–50 = non-alexithymic subjects; 51–60 = borderline alexithymic subjects; 61–100 = alexithymic subjects[2]. In contrast to the BVAQ, the TAS-20 scale focuses only on the cognitive dimension of the alexithymia[3].

Odor awareness Scale. The questionnaire is a measure of people's self- assessments of their tendency to notice, pay attention, or attach importance to odors in certain everyday situations, and

their knowledge of how olfactory perception shape their behaviors. It is composed by 32 items with five-point scales used as response categories in most cases. The total score is obtained by adding up the scores of the individual items, and can range between 32 and 158, with higher scores indicating greater odor awareness[4].

Vividness olfactory imagery. The VOIQ[5] questionnaire requires participants to mentally evoke a series of 16 objects and activities that have an odorous element and to rate the realism and the vividness of each of the imagined on a 5-point scale (from 1 "perfectly vivid" to 5 "no image at all"). The score of VOIQ ranges from 16 to 80 (low scores indicating good imaging ability).

Odors selection. Before the start of the experimental study, a first pilot study was carried out to select appropriate odor stimuli. 7 odors, selected from the literature (e.g. [6-8]), diluted in mineral oil or propylene glycol, were rated by 11 normosmic participants (not included in this experimental study) for intensity, pleasantness and familiarity (see Table 1S and Supplemental Information for raw data). Ratings were collected on a 10-cm paper designed VAS, ranging from "not at all" to "very much". Odors were presented manually through jars. Jars were distinguished from one another only through numbers. We considered pleasantness ratings to select one unpleasant odor, on pleasant odor and one neutral odor. In addition, we also avoided to select stimuli that resulted very intense or very familiar (scores > 7). See table 1S.

Table 1S. Means (standard deviations) of odor ratings in Pilot 1.

	Gardenia essential oil	Isovaleric Acid	Lavender Oil	Butyric Acid	Cedarwood Oil	Grapefruit essential oil	Tangerine essential oil
	**Mean (SD)**	**Mean (SD)**	**Mean (SD)**	**Mean (SD)**	**Mean (SD)**	**Mean (SD)**	**Mean (SD)**
Pleasantness	8.09 (2.17)	1.79 (1.48)	7.96 (1.14)	1.82 (0.80)	4.99 (2.76)	7.66 (2.20)	8.20 (1.28)
Intensity	4.87 (2.08)	7.36 (1.56)	7.04 (1.29)	4.90 (2.31)	2.09 (2.59)	7.56 (1.15)	8.06 (1.08)
Familiarity	6.67 (2.67)	5.09 (2.10)	8.36 (1.05)	5.42 (3.17)	2.67 (2.36)	7.19 (2.58)	8.08 (2.61)

Afterwards, 45 normosmic participants, recruited for another study[9] but excluded from the present experimental study, were asked to rate again these odors for intensity, pleasantness and familiarity. Similarly, ratings were collected on a 10-cm computerized VAS, ranging from "not at all" to "very much." but in this pilot study odors were presented though a computer controlled-olfactometer. This pilot was carried out to assure that the presentation of odors through olfactometer would have not change their pleasantness. Statistical analyses (see below) confirmed the results of the first pilot study.

For intensity rating (neutral odor: M = 7.00, SD = 1.96; pleasant odor: M = 4.497, SD = 1.96; unpleasant odor: M = 4.13, SD = 1.73), the LMM with odors as factors showed that neutral odor was rated as more intense than pleasant (ß = -2.53, SE = 0.69, $t = -3.67$, $p < .001$) and unpleasant odors (ß = -2.87, SE = 0.69, $t = -4.16$, $p < .001$). For pleasantness rating (neutral odor: M = 4.27, SD = 1.67; pleasant odor: M = 5.73, SD = 1.83; unpleasant odor: M = 3.00, SD = 1.60), the

For pleasantness, LMM with odors as factors showed a significant effect for odor condition: pleasant odor was rated as significantly more pleasant than neutral odor (ß = 1.46, SE = 0.62, $t = 2.36$, $p = .002$) and unpleasant odor was rated as significant more unpleasant than neutral odor (ß = -1.27, SE = 0.62, $t = -2.03$, $p = .048$). Finally for familiarity ratings (neutral odor: M = 6.14, SD = 2.18; pleasant odor: M = 4.53, SD = 1.73; unpleasant odor: M = 2.73, SD = 1.49) the LMM with odors as factors showed that neutral odor was rated as more familiar than pleasant (ß = -1.61, SE = 0.67, $t = -2.39$, $p = .02$) and unpleasant odors (ß = -3.41, SE = 0.67, $t = -5.07$, $p < .001$).

Importantly, since participants enrolled in the main experimental study did not rate gardenia as a pleasant odor and since several concerns were raised regarding the use of cedarwood oil (see for instance[10]), we used gardenia as a neutral odor. The inconsistency between the odor pilot and the main experiment regarding gardenia ratings could be due to the different experiment design. While in the second pilot, each odor was presented once followed by the three questions (i.e. intensity, pleasantness and familiarity); in the main experiment, each odor was presented three times (in one single block) and each presentation was followed by only one question. This experimental

procedure could have increased the amount of odor presented in the final presentation enhancing the perception of odor intensity and decreasing the pleasantness of the odor. In addition, even if we carefully controlled odor presentation, we cannot exclude the effects of odor contamination between blocks.

Supplemental results

Table 2S. Means (standard deviations) of odor ratings

Measure	Group	Clean Air	Neutral	Unpleasant
**Odor Ratings**				
**Intensity**				
	LA	2.71 (2.15)	6.62 (2.38)	7.57 (2.62)
	MA	2.79 (1.99)	6.85 (1.53)	7.50 (1.85)
	HA	2.70 (1.95)	6.95 (2.40)	6.95 (2.97)
**Pleasantness**				
	LA	4.65 (2.46)	3.86 (2.10)	2.52 (2.46)
	MA	4.58 (1.43)	4.60 (2.21)	1.95 (1.28)
	HA	4.05 (2.18)	4.38 (2.22)	1.70 (0.80)
**Familiarity**				
	LA	3.05 (2.15)	5.52 (2.18)	6.45 (3.63)
	MA	2.72 (1.78)	5.30 (2.08)	4.95 (2.44)
	HA	2.84 (1.83)	5.43 (2.52)	6.57 (3.03)
**Reaction Times (ms)**				
**Intensity**				
	LA	2518.33 (711.14)	2527.86 (816.20)	2430.52 (1095.75)
	MA	2340.74 (560.18)	2642.30 (689.54)	2327.90 (856.69)
	HA	2404.35 (925.78)	2583.76 (517.07)	2419.29 (793.89)
**Pleasantness**				
	LA	2978.75 (1132.46)	2820.38 (940.53)	2119.38 (686.48)
	MA	2990.00 (1200.13)	2779.15 (771.00)	2183.95 (849.74)
	HA	2844.90 (864.22)	2540.71 (959.71)	2443.90 (718.04)
**Familiarity**				
	LA	3286.32 (1160.45)	2765.14 (1145.45)	2427.15 (868.96)
	MA	3125.33 (914.01)	2444.35 (766.16)	2637.95 (751.68)
	HA	2709.11 (1278.35)	2683.00 (786.15)	2843.19 (1189.20)

Odor rating task: reaction times. For reaction times (RTs) LMM with only odor factor resulted the best model. LMM on RTs of intensity rating (AIC = 2945.90; BIC = 2961.95; logLik = -1467.95; R^2 = 0.11) revealed odor as significant factor (clean air: M = 2424.10ms, SD = 740.74;

neutral odor: M = 2583.71ms, SD = 675.56; unpleasant odor: M = 2340.72ms, SD = 817.63; see figure 2) however no significant differences were found between clean air, neutral and unpleasant odors (all ß < 2340.13, all SE > 96.56, all $t < 24.48$, all $p > .06$). The LMM model including the interaction group*odor factors showed no significant differences between groups and no significant interactions.

LMM on RTs of pleasantness rating (AIC = 2984.09; BIC = 3000.11; logLik = -1487.05; $R^2 = 0.28$) revealed odor as significant factor (clean air: M = 2884.05ms, SD = 983.82; neutral odor: M = 2712.35ms, SD = 890.39; unpleasant odor: M = 2246.95ms, SD = 754.29; see figure 2): participants were faster for unpleasant odor compared to other odors (all ß > 469.55, all SE < 140.79, all $t > 3.33$, all $p < .001$). No significant differences were found between clean air and neutral odor (ß = -186.36, all SE = 142.20, all $t < -1.31$, all $p > .19$). The LMM model including the interaction group*odor factors showed no significant differences between groups and no significant interactions.

LMM on RTs of familiarity rating (AIC = 2911.44; BIC = 2927.29; logLik = -1450.72; $R^2 = 0.22$) revealed odor as significant factor (clean air: M = 3038.73ms, SD = 1137.95; neutral odor: M = 2633.84ms, SD = 913.34; unpleasant odor: M = 2639.49ms, SD = 958.64; see figure 2): however no significant differences were found between clean air, neutral, and unpleasant odors (all ß < -2648.88, all SE > 120.94, all $t < -0.09$, all $p > .12$). The LMM model including the interaction group*odor factors showed no significant differences between groups and no significant interactions.

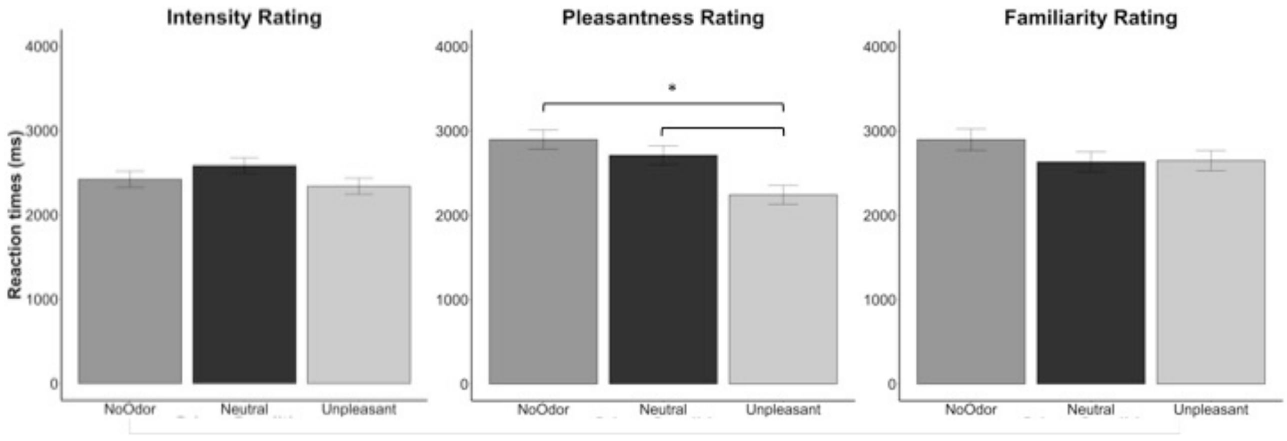

Figure 1S. Distribution of reaction times of intensity, pleasantness, familiarity ratings per odor conditions. Error bars represent the simulated 95% confidence interval of the coefficients. Significant differences are indicated.

Odor-rating task: SCR controlling for BDI. LMM with BDI and interaction between odor and group resulted the best model fitting SCR (AIC = -251.75; BIC = -203.12; logLik = 137.87; R^2 = 0.38). The analysis showed the same result we found without BDI. We then run three analyses with only odor and BDI as factors for each group. LA group showed greater SCR for neutral (ß = 0.08, SE = 0.04, t = 2.26, p = .002) and unpleasant odors (ß = 0.07, SE = 0.03, t = 2.09, p = .04) compared to clean air. No significant differences were found between neutral and unpleasant odors (all ß < 0.01, all SE > 0.03, all t < 0.51, all p > .61). No significant differences were found between odors for HA (all ß < 0.005, all SE > 0.03, all t < 0.16, all p > .76) and for MA (all ß < 0.004, all SE > 0.03, all t < 1.46, all p > .14). BDI factor showed a significant effect for LA group (ß = 0.01, SE = 0.004, t = 2.28, p = .003) and for HA group (ß = -0.01, SE = 0.004, t = -2.84, p = .01) with opposite effects: in the LA group the more participants were depressed the greater the SCR, in the HA group the more participants were depressed the weaker the SCR.

Odor-rating task: IHR controlling for BDI. LMM with BDI and interaction between time windows and group resulted the best model fitting IHR (AIC = 11816.34; BIC = 11881.16; logLik = -5896.17; $R^2 = 0.97$). BDI factor resulted no significant confirming the results of previous analyses.

Correlation between BVAQ and TAS-20 questionnaires. The TAS-20 questionnaire was significant correlated with the total score of BVAQ ($r = 0.55$, $p < .0001$, 95% CI [0.35, 0.70]). Moreover, as expected, it was significant correlated with the subscales that composed the cognitive dimension: B1 ($r = 0.54$, $p < .0001$, 95% CI [0.34, 0.69]), B3 ($r = 0.40$, $p < .0001$, 95% CI [0.17, 0.59]) and B5 ($r = 0.47$, $p < .0001$, 95% CI [0.24, 0.64]); but it TAS-20 was not correlated with the subscales that composed the affective dimension of BVAQ (B2 and B4).

References

1 Bermond, B. *et al.* A cognitive and an affective dimension of alexithymia in six languages and seven populations. *Cognition and Emotion* **21**, 1125-1136 (2007).

2 Bressi, C. *et al.* Cross validation of the factor structure of the 20-item Toronto Alexithymia Scale: an Italian multicenter study. *Journal of Psychosomatic Research* **41**, 551-559 (1996).

3 Bermond, B., Bierman, D. J., Cladder, M. A., Moormann, P. P. & Vorst, H. C. The cognitive and affective alexithymia dimensions in the regulation of sympathetic responses. *International Journal of Psychophysiology* **75**, 227-233 (2010).

4 Smeets, M. A., Schifferstein, H. N., Boelema, S. R. & Lensvelt-Mulders, G. The Odor Awareness Scale: a new scale for measuring positive and negative odor awareness. *Chemical senses* **33**, 725-734 (2008).

5 Gilbert, A. N., Crouch, M. & Kemp, S. E. Olfactory and visual mental imagery. *Journal of Mental Imagery* (1998).

6 Boesveldt, S., Frasnelli, J., Gordon, A. & Lundström, J. The fish is bad: Negative food odors elicit faster and more accurate reactions than other odors. *Biological psychology* **84**, 313-317, doi:10.1016/j.biopsycho.2010.03.006 (2010).

7 Delplanque, S. *et al.* Emotional processing of odors: evidence for a nonlinear relation between pleasantness and familiarity evaluations. *Chemical Senses* **33**, 469-479 (2008).

8 Alaoui-Ismaili, O., Vernet-Maury, E., Dittmar, A., Delhomme, G. & Chanel, J. Odor hedonics: connection with emotional response estimated by autonomic parameters. *Chemical senses* **22**, 237-248 (1997).

9 Cecchetto, C., Rumiati, R. & Parma, V. Relative contribution of odour intensity and valence to moral decisions. *Perception* **Epub** (2017).

10 Dayawansa, S., Umeno, K., Takakura, H., Hori, E., Tabuchi, E., Nagashima, Y., ... & Nishijo, H. Autonomic responses during inhalation of natural fragrance of “Cedrol” in humans. *Autonomic Neuroscience*, 108(1), 79-86 (2003).
